# Solar-Driven Rhodamine B Degradation Using Biogenically Recovered Mixed Metal(Loid) Sulfides Derived from Metallurgical Waste

**DOI:** 10.3390/ijms27135689

**Published:** 2026-06-24

**Authors:** María Rosario Sánchez-Macías, Adrián Ramírez Parada, Diego Hernández Martinez, Santos J. Castillo, Francisco J. Almendariz Tapia, Francisco J. Cervantes, Aurora M. Pat-Espadas

**Affiliations:** 1 Department of Chemical and Metallurgical Engineering, Interdisciplinary Faculty of Engineering, University of Sonora, Hermosillo 83000, Mexico; 2 Department of Physics Research, University of Sonora, Blvd. Luís Encinas y Rosales S/N Centro, Hermosillo 83000, Mexico; 3 Laboratory for Research on Advanced Processes for Water Treatment, Engineering Institute, Campus Juriquilla, Universidad Nacional Autónoma de México (UNAM), Blvd. Juriquilla 2001, Querétaro 76230, Mexico

**Keywords:** photocatalysis, metal(loid) sulfides, wastewater treatment, resource recovery, circular economy

## Abstract

Biogenically recovered mixed metal(loid) sulfides (BPS) obtained from metallurgical effluents were evaluated as sustainable photocatalysts for the solar-driven degradation of Rhodamine B (RhB). The material, recovered using biogenic sulfide produced by sulfate-reducing bacteria in an upflow anaerobic sludge bed reactor, was mainly composed of Sb_2_S_3_ and Bi-containing sulfide phases and exhibited a fibrous morphology and a narrow direct band gap of 1.306 eV. Under solar irradiation, BPS achieved RhB degradation efficiencies above 98% under the evaluated conditions (0.8 g L^−1^ catalyst and 5 mg L^−1^ dye), consistently outperforming reagent-grade Sb_2_S_3_. Photocatalytic degradation followed pseudo-first-order kinetics (R^2^ > 0.90), and the apparent reaction rate constant was more than five times higher than that of the reference material under the best-performing conditions. A preliminary reusability assessment and post-reaction characterization after three photocatalytic cycles revealed no significant morphological or compositional changes in BPS. These results demonstrate that waste-derived metal(loid) sulfides recovered through a biogenic process can serve as effective solar photocatalysts, highlighting a promising circular-economy strategy for transforming metallurgical residues into value-added materials for water remediation.

## 1. Introduction

The presence of organic pollutants in wastewater has increased in recent years due to the widespread use of synthetic organic compounds across various industrial sectors coupled with the limited efficiency of conventional water treatment processes [1,2]. Among these contaminants, dyes, pharmaceuticals, personal care products, fertilizers and pesticides are of particular concern due to their persistence and potential environmental impact [3]. Although conventional treatment technologies can remove certain contaminants, their application to pollutants with complex molecular structures is often limited by reduced treatment efficiency, high operational costs, and process complexity [4].

In this context, photocatalytic degradation has emerged as a promising alternative for the treatment of organic contaminants in wastewater, as it enables the transformation of recalcitrant compounds into less harmful products under light irradiation [5,6,7].

Beyond pollutant degradation, some studies have demonstrated the strong link between photocatalysis and sustainable metal recovery. Photocatalytic systems have been used to selectively dissolve, separate, and recover precious metals such as gold and palladium from waste streams and electronic waste through the action of photogenerated reactive species. Research has progressed from elucidating the fundamental mechanisms of photocatalytic metal dissolution to developing scalable approaches for precious metal recycling, highlighting photocatalysis as a promising technology for urban mining and circular economy applications [8,9,10,11].

Among the available photocatalysts, Sb_2_S_3_ has attracted increasing attention due to its narrow band gap (1.5–2.5 eV), which enables efficient absorption across the visible and near-infrared regions of the spectrum, as well as its demonstrated potential for the degradation of organic pollutants [12,13,14,15]. Consequently, significant efforts have been devoted to the development of Sb_2_S_3_-based materials, evolving from bulk systems toward nanostructures and heterojunctions designed to improve charge separation and photocatalytic efficiency [12,13,16]. Morphology engineering has proven particularly effective, as nanostructured Sb_2_S_3_ materials, including nanospheres and nanorods, exhibit enhanced charge transport and degradation efficiencies ranging from 80% to 96% under irradiation conditions [17,18]. In addition, heterojunctions based on Sb_2_S_3_, including composites with carbonaceous materials and Bi-containing semiconductors, have shown superior photocatalytic performance owing to improved charge separation, reduced electron–hole recombination, and extended light absorption ranges [19,20,21].

In particular, Bi_2_O_3_/Sb_2_S_3_ heterojunctions have demonstrated remarkable photocatalytic activity under visible and solar irradiation while exhibiting enhanced stability and interfacial electron transfer compared with their individual components [20]. The improved performance has been mainly attributed to more efficient charge separation, reduced electron–hole recombination, and an extended light absorption range resulting from the synergistic interaction between both semiconductors [20]. Nevertheless, bismuth-based photocatalysts still face challenges associated with rapid charge recombination, limited interfacial charge-transfer efficiency, and long-term operational stability, which continue to motivate the development of advanced heterojunctions, cocatalysts, and defect-engineering strategies [19,20,22,23,24].

Despite the promising performance of Sb_2_S_3_-based heterojunctions and related photocatalysts, most reported materials are produced through conventional synthetic routes that often involve complex procedures, high energy consumption, and additional environmental burdens [21,25]. In contrast, the recovery of sulfide-based materials through biogenic processes, such as sulfate-reducing systems, offers a sustainable pathway for transforming metallurgical effluents into value-added functional materials [26]. Unlike highly controlled synthetic photocatalysts, materials recovered from real effluents are inherently polymetallic and may contain mixed sulfide phases such as Sb_2_S_3_, Bi_2_S_3_, and As_2_S_3_ [26]. The chemical form in which metals or metalloids are recovered plays a key role in determining their optical, electronic, and structural properties, which in turn govern their functional performance. These characteristics are directly linked to the electronic structure, charge density distribution, and bonding environment, ultimately defining their reactivity and applicability in catalysis, energy, and environmental remediation [27]. Consequently, the coexistence of multiple sulfide phases with diverse morphologies and electronic interactions may generate synergistic effects that contribute to photocatalytic activity. Nevertheless, the photocatalytic potential of these biogenically recovered polymetallic sulfides remains largely unexplored.

Among target contaminants, Rhodamine B is a widely used synthetic dye characterized by high chemical stability and environmental persistence. Its release into water bodies can cause toxic effects on aquatic organisms and disrupt biological processes even at low concentrations [28,29,30,31]. Furthermore, its physicochemical properties favor adsorption and accumulation in soils, facilitating bioaccumulation in crops and posing risks to human health through bioavailability, organ accumulation, and interactions with DNA [32].

In this work, the photocatalytic activity of biogenically recovered mixed metal(loid) sulfides obtained from a real metallurgical effluent was evaluated for the degradation of Rhodamine B (RhB) under solar light irradiation. Emphasis was placed on understanding how the structural, optical, and compositional properties of the recovered polymetallic phases drive their photocatalytic performance in comparison with reagent-grade Sb_2_S_3_. To this end, detailed physicochemical characterization was conducted before and after photocatalytic operation to evaluate structure–property–function relationships and material preservation after repeated photocatalytic use.

This study provides new insights into the use of waste-derived mixed metal(loid) sulfides as sustainable photocatalysts, contributing to integrated strategies for metallurgical waste valorization and solar-driven environmental remediation.

## 2. Results and Discussion

### 2.1. Characterization of the Biogenically Recovered Mixed Metal(Loid) Sulfides (BPS)

#### 2.1.1. Textural Characterization by Nitrogen Physisorption (BET)

BET analysis was used to determine the textural properties reagent-grade Sb_2_S_3_ (RG-Sb_2_S_3_) and the biogenically recovered mixed metal(loid) sulfides (BPS). The obtained values of surface area, total pore volume, and average pore diameter values are summarized in Table 1.

RG-Sb_2_S_3_ exhibited a low surface area of 0.34 m^2^/g and a pore volume of 0.0002 cm^3^/g. In contrast, BPS showed an increased surface area of 2.99 m^2^/g, while the pore volume remained low (0.0001 cm^3^/g).

Pore size distribution analysis (Figure S1) indicated the presence of both micropores (1.7–2.0 nm) and mesopores (~2.1–2.5 nm) in BPS. This can be attributed to the precipitation conditions during material synthesis and the possible formation of structural voids associated with the biological environment [25,33]. The predominance of pores in the small mesoporous range, together with a minor contribution of micropores, may enhance the accessibility of reactants to surface active sites while facilitating mass transfer within the material. For Rhodamine B, the presence of mesopores is particularly relevant because it can improve molecular diffusion and reduce transport limitations commonly associated with highly microporous structures [21,25]. Consequently, the combined micro–mesoporous structure of BPS may contribute to both contaminant adsorption and subsequent photocatalytic degradation.

#### 2.1.2. XRD Analysis

**Figure 1:**
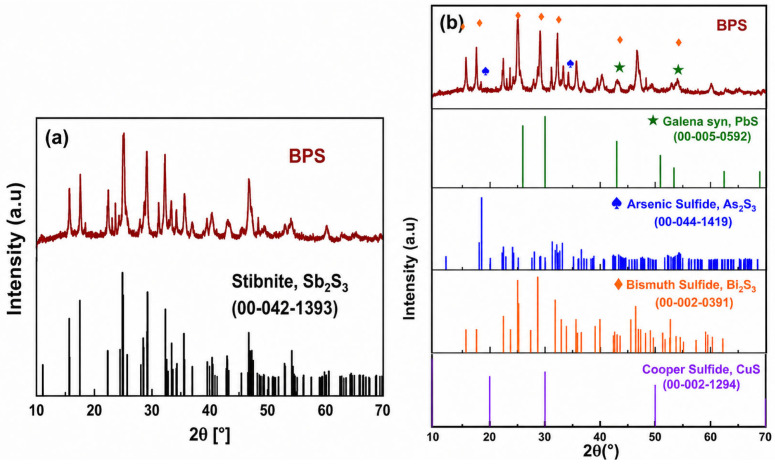
XRD patterns of the biogenically recovered mixed metal(loid) sulfides (BPS) and reference diffraction patterns corresponding to (**a**) stibnite and (**b**) other metal/metalloid sulfide phases.

The diffractogram presented in Figure 1 exhibits well-defined, high-intensity peaks that closely match the characteristic reflections of stibnite (Sb_2_S_3_), in agreement with JCPDS card 00-042-1393. These results indicate that crystalline Sb_2_S_3_ is the predominant phase in the solids; however, the presence of additional peaks suggests the coexistence of other phases, such as Bi_2_S_3_. Considering the presence of multiple metals and metalloids, the XRD patterns were further compared with reference patterns of their corresponding sulfides, indicating that the material likely consists of a mixture of mixed metal(loid) sulfides.

Elemental analysis of the BPS, previously reported in [26], showed that the recovered mixed metal(loid) sulfides were mainly composed of Sb (68.8%), followed by Bi (21%), As (8.7%), Pb (1.2%), and Cu (0.31%). The marked enrichment of Sb within the matrix was evidenced by concentrations approximately three, eight, and 57 times greater than those of Bi, As, and Pb, respectively, and more than 200-fold higher than Cu [26].

Although multiple sulfide phases (Sb–Bi–As–Pb–Cu) were identified, such compositional complexity may enhance functional properties, particularly for photocatalytic applications, due to the potential increase in active sites and improved charge separation reported for mixed or bismuth-containing systems [34,35,36]. Therefore, obtained BPS can be considered a promising candidate for photocatalytic applications in environmental remediation under solar irradiation.

#### 2.1.3. FTIR Characterization

**Figure 2:**
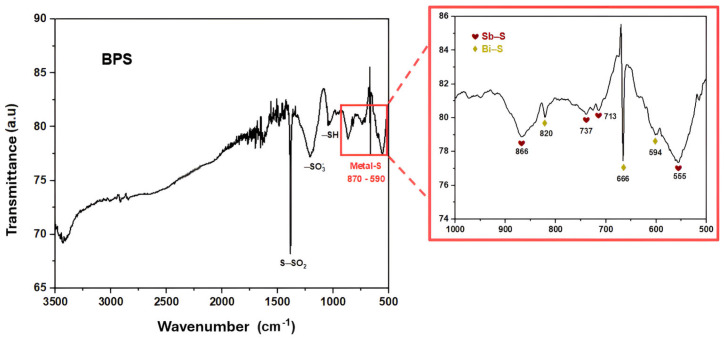
FTIR spectra of the biogenically recovered mixed metal(loid) sulfides material (BPS).

FTIR spectroscopy was used to investigate the chemical structure of the biogenically recovered mixed metal(loid) sulfides (BPS); the spectra are shown in the range of 3500 to 500 cm^−1^ in Figure 2.

The spectrum presents absorption bands between 876 and 590 cm^−1^, attributable to sulfur stretching vibrations and metal–sulfur (M–S) bonds, characteristic of Sb, Bi, As, Pb, and Cu sulfides [37,38,39]. Although the typical Sb–S peaks (713, 737–740, 866 cm^−1^) are not clearly observed, the signals at 668 and 593 cm^−1^ could be related to such bonds, considering possible modifications of the chemical environment induced by the biological precipitation process [21,40].

Additionally, the bands at 594, 666 and 820 cm^−1^ indicate the possible presence of Bi–S bonds, while the signals at 1382, 1242 and 1035 cm^−1^ are associated with bending vibrations of the N–H group (amide III), stretching of sulfonates (–SO_3_^−^) and residual thiols (S–SO_2_, –SH), evidencing the compositional complexity of BPS [38,41].

FTIR analysis confirms the formation of metal and metalloid sulfides with predominant M–S bonds and suggests the presence of diverse chemical interactions that could drive the functional properties of the material, particularly in photocatalytic applications.

#### 2.1.4. SEM-EDS Analysis

**Figure 3:**
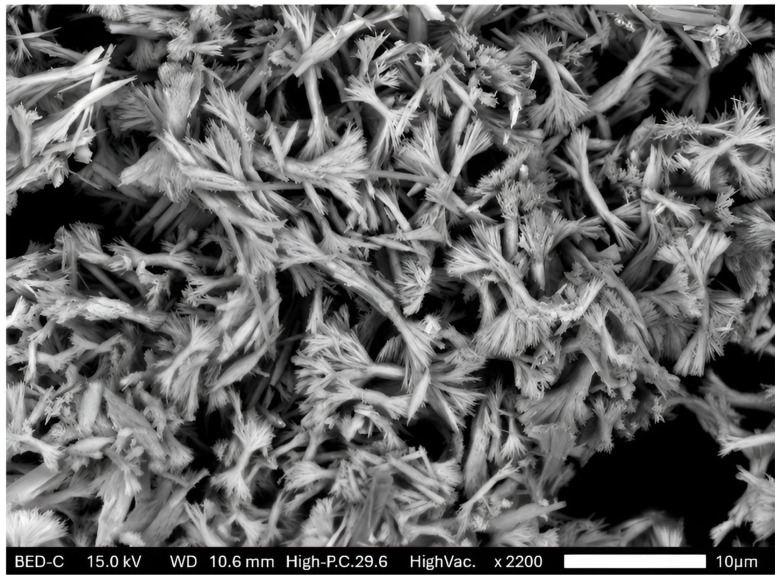
SEM image of biogenically recovered polymetallic sulfide material (BPS).

The morphology reveals that the material exhibits an acicular and fibrillar crystalline structure, consisting of a central axis from which multiple fine needle-like or fiber-like branches emerge (Figure 3). Morphometric measurements were performed on 146 structures: the average total crystal length was 7.40 ± 0.97 μm, while the stems measured 2.38 ± 0.48 μm and the fine branches 2.58 ± 0.64 μm (Figure S2, Table S1).

Considering the two-stage process from which the material was obtained, involving recovery from metallurgical effluent using biogenic sulfide, the imposed controlled conditions may promote preferential crystal growth, leading to the formation of well-defined crystalline phases. Although the synthesis conditions differ from those reported in the literature obtained by other synthesis methods, some mechanistic insights may still be relevant for comparison.

A possible formation mechanism involves an initial nucleation stage in which small particles act as seeds that subsequently aggregate to form a larger central core, as described by [42]. During growth, these seeds may develop along preferential directions through a solid–solution–solid (SSS) transformation mechanism, potentially leading to anisotropic structures.

Under such conditions, the sequential formation and attachment of rod-like units, influenced by Sb–S chain anisotropy and non-equilibrium growth, may contribute to the development of complex superstructures. However, given the differences in synthesis approach, these interpretations should be considered cautiously. The exact formation mechanism of the nanorod-based superstructures obtained in this study remains unclear and warrants further investigation.

For instance, precursor concentration has also been identified as a key factor influencing morphology. Previous studies report that increasing precursor concentration leads to a higher formation of Sb_2_S_3_ submicrometer-sized rod bundles. Thus, achieving a high yield of bundle-like stibnite structures requires relatively high precursor concentrations to enhance both nucleation and growth rates [43].

Consistent with this behavior, the BPS employed in this study was obtained from batch precipitation tests using a metallurgical effluent diluted to an initial Sb concentration of 1000 mg/L in batch precipitation tests. Under these conditions, an operational yield of 37 g of solids per liter of treated effluent was obtained, together with Sb precipitation efficiencies of up to 95.4% [26].

Reaction time is another critical parameter governing crystal growth. Earlier studies have shown the evolution of stibnite crystals from one-dimensional submicrometer-sized rods into straw-like morphologies over time. These observations demonstrate that nucleation and growth processes occur rapidly, allowing fully developed bundles to form within 20–40 min [43]. The 24 h precipitation time used to obtain the material may have also induced both the formation of micrometer-sized structures and the observed morphology.

The presence of hierarchical structures, such as nanoflower-like or hedgehog-shaped aggregates, is consistent with previous reports on similar materials such as Sb_2_S_3_/SiO_2_ and Bi_2_S_3_/Bi_2_WO_6_, where the particles adopt uniformly connected rod configurations, reflecting control over the nucleation and crystal growth processes [36,44].

Further analysis by energy-dispersive X-ray spectroscopy (EDS) (Figure S3) confirmed the presence of Sb, Bi, and S in the biogenically recovered mixed metal(loid) sulfides (BPS). Quantitative analysis showed Sb (31.72 ± 0.81 wt%), Bi (10.18 ± 1.40 wt%), and S (19.03 ± 0.84 wt%). These results support the formation of Sb_2_S_3_ as the main phase, in agreement with the XRD results.

**Figure 4:**
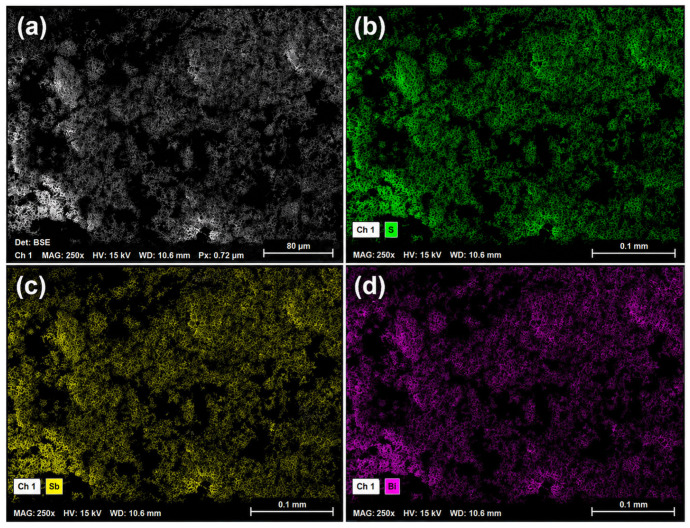
EDS elemental mappings (**a**) SEM image BPS. (**b**) Corresponding EDS element mapping of S (**c**), Sb and Bi (**d**).

EDS elemental mapping revealed a homogeneous distribution of Sb, Bi, and S throughout the material (Figure 4), indicating a uniform integration of the elements derived from the original metallurgical effluent. This morphology and hierarchical organization may favor charge separation and enhance interaction with contaminants, which are relevant features for photocatalytic applications. Similar hierarchical architectures, such as CuWO_4_/Bi_2_S_3_ and ZIF-67, have been reported to exhibit improved functional performance due to their surface area and efficient charge transfer pathways [36,45,46].

#### 2.1.5. Determination of BPS Band Gap

The optical properties and absorption range of BPS were evaluated by diffuse reflectance spectroscopy (DRS) in the 200–800 nm range. The band gap (*E*_g_) was determined from linear extrapolation from Tauc plots [47,48,49,50], considering direct (*n* = 1/2) and indirect (*n* = 2) transitions, in agreement with previous reports for Sb_2_S_3_ [45,51,52,53]. The data and linear fitting parameters used for the construction of the tangent lines employed in the band gap determination are provided in Tables S2 and S3.

**Figure 5:**
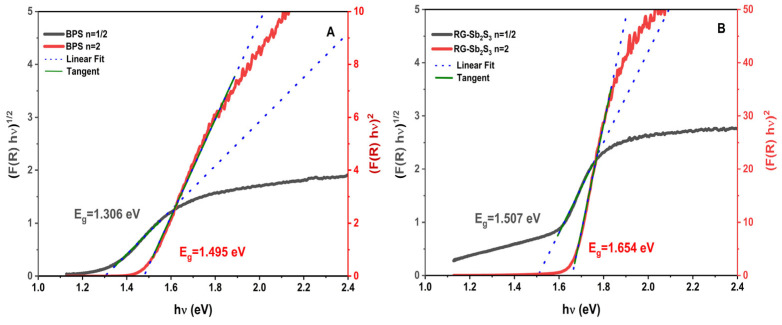
Determination of the band gap energy: (**A**) BPS. (**B**) RG-Sb_2_S_3_.

The biogenically recovered mixed metal(loid) sulfides (BPS) exhibited band gap values of 1.306 eV (direct transition) and 1.491 eV (indirect transition), while the reagent-grade Sb_2_S_3_ (RG-Sb_2_S_3_) showed values of 1.507 eV and 1.654 eV, respectively (Figure 5).

The lower apparent band gap observed for BPS relative to RG-Sb_2_S_3_ may be related to the polymetallic nature of the recovered material. XPS and phase characterization revealed the coexistence of Sb- and Bi-containing sulfide species, suggesting the presence of chemically distinct semiconducting domains and interfacial regions within the BPS matrix. Similar Bi- and Sb-based sulfide heterostructures have been reported to promote charge separation and improve photocatalytic activity through interfacial charge-transfer processes [54,55]. Depending on the band alignment and electronic structure of the constituent phases, these interactions have been interpreted as Type-II, Z-scheme, or S-scheme heterojunctions in related systems [56,57,58]. Therefore, the coexistence of multiple sulfide phases in BPS may influence charge-carrier dynamics and contribute to the enhanced photocatalytic performance observed. However, additional studies are required to elucidate the specific electronic interactions and charge-transfer mechanisms operating within the BPS matrix.

Antimony sulfide (Sb_2_S_3_) and related metal sulfides have been extensively investigated as photocatalysts due to their favorable optical and electronic properties. Their relatively narrow band gaps, typically ranging from 1.0 to 2.5 eV, enable efficient absorption of visible light and make them attractive materials for solar-driven applications [59,60].

In this context, the band gap values obtained for BPS fall within the visible-light region, supporting its ability to utilize solar irradiation and highlighting its potential as a photocatalyst. The reduced band gap relative to RG-Sb_2_S_3_ may broaden the absorption range and, together with the coexistence of multiple sulfide phases, contribute to the improved photocatalytic performance of the biogenic material [21,25,44].

#### 2.1.6. XPS

Figure S4 shows the XPS survey spectrum of BPS, confirming the presence of Sb, S, Bi, O, C and N on its surface. The presence of dominant Sb and S signals indicates a surface mainly composed of Sb-S compounds, consistent with Sb_2_S_3_ formation.

Multiple components observed in the Sb and S regions indicate the coexistence of different chemical environments, reflecting the heterogeneous surface chemistry typically associated with polymetallic metallurgical-derived materials [61,62].

The Sb 3*d* doublet (3*d*_5_/_2_ and 3*d*_3_/_2_) suggest the +3-oxidation state of antimony, supporting the presence of Sb_2_S_3_. Additionally, shifts toward higher binding energies indicate the presence of oxidized antimony species, such as Sb_2_O_5_, likely formed due to air exposure. In the sulfur region, S 2*p* signals typical of stibnite are identified, while the O 1*s* signal suggests the presence of surface oxides or absorbed species such as water or carbonates.

**Figure 6:**
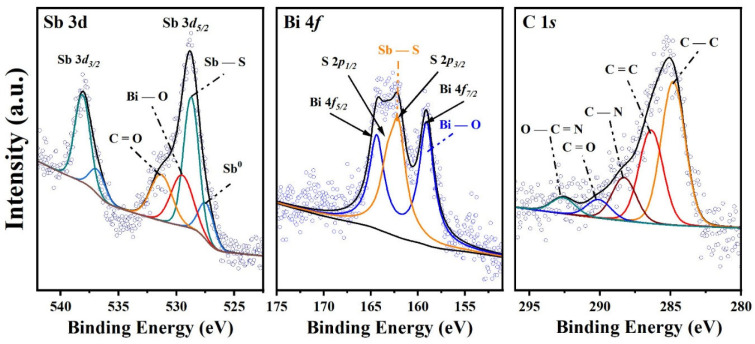
Deconvolution of XPS spectra of polymetallic sulfide material (BPS) in different regions: Sb 3*d*, Bi 4*f* and C 1*s*.

Figure 6 shows the high-resolution XPS spectra of the Sb 3*d*, Bi 4*f*, and C 1*s* core levels for the BPS sample, providing information on the chemical states and surface composition of the BPS material, following oxidation-state analysis methodologies reported in recent literature [63].

The high-resolution Sb 3*d* spectrum exhibits a characteristic spin–orbit doublet associated with Sb 3*d*_5/2_ and Sb 3*d*_3/2_ components, with an energy separation of approximately Δ = 9.4 eV. The main component contribution located at ~528.8 eV is assigned to Sb-S bonds, confirming the presence of antimony sulfide species within the material surface. A low-intensity component contribution located at ~527.7 eV is attributed to metallic antimony (Sb^0^), suggesting the presence of a small amount of unreacted antimony or reduced surface species. In addition, the Sb 3*d* spectra overlaps O 1*s* signal. The components observed at ~529.5 and ~531.5 eV is attributed to metal-oxygen and carbonyl-related species, respectively, associated with Bi-O and C=O bonding. These contributions indicate the presence of the Bi_2_O_3_ precursor and a small amount of organic contamination on the BPS surface.

The Bi 4*f* spectrum exhibits the characteristic spin–orbit doublet of Bi 4*f*_7/2_ and Bi 4*f*_5/2_ with an energy separation of approximately Δ = 5.3 eV. The dominant peaks located at ~159 eV and 164.3 eV are assigned to Bi–O bonds, confirming the presence of bismuth in an oxidized chemical state. A secondary contribution from the S 2*p* overlap signal, located at ~162 eV corresponds to Sb-S bonding, confirming the presence of a Sb_2_S_3_ at the surface of the material. The coexistence of Bi-O and Sb-S confirms the successful incorporation of both antimony sulfide species and bismuth oxide species in the BPS compound.

The high-resolution C 1*s* spectrum was deconvoluted into several components corresponding to different carbon environments. The main peak located at approximately ~284.8 eV is assigned to C-C bonds, while the contribution at slightly higher binding energies is assigned to C=C species. Additional components centered near ~286 eV and ~288 eV is associated with C-N and C=O bonds, respectively. A weak contribution at higher binding energy is assigned to O-C=N species. These carbon-related signals are commonly attributed to residual organic compounds from the biogenic recovery process and adventitious carbon adsorbed on the sample surface during handling and exposure to air.

Overall, the XPS results confirm the coexistence of an antimony sulfide and bismuth oxide species in the BPS material, as evidenced by the predominant Sb-S and Bi-O chemical states. The oxygen-related contributions are mainly associated with bismuth oxide and surface oxidation, whereas the carbon-related species originate from residual organic compounds and atmospheric contamination.

### 2.2. Photocatalytic Performance and Degradation Efficiency

#### 2.2.1. Photocatalytic Degradation of Rhodamine B: Reaction Kinetics and Fitting to Model

**Figure 7:**
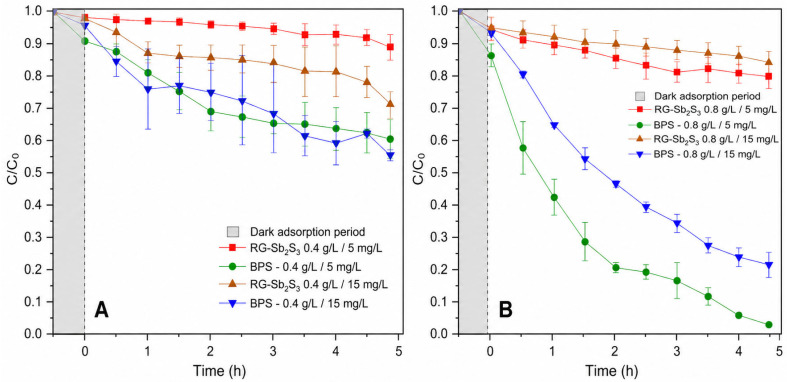
Photocatalytic degradation of the materials under solar irradiation: (**A**) kinetics at 0.4 g/L; (**B**) material behavior at a dose of 0.8 g/L.

The photocatalytic degradation of Rhodamine B was evaluated using the biogenically recovered mixed metal(loid) sulfides (BPS) and reagent-grade Sb_2_S_3_ (RG-Sb_2_S_3_) under solar irradiation (Figure 7). The results show that BPS exhibited higher photocatalytic activity than RG-Sb_2_S_3_ across the tested conditions. At a fixed photocatalyst loading of 0.4 g/L (Figure 7A) and an initial Rhodamine B concentration of 5 mg/L, BPS achieved 38.98% degradation, compared to 10.23% for RG-Sb_2_S_3_. At 15 mg/L, degradation efficiency increased for both materials, reaching 44.20% for BPS and 27.80% for RG-Sb_2_S_3_. Increasing the catalyst loading to 0.8 g/L (Figure 7B) substantially enhanced the performance of BPS, resulting in more than 98% degradation at 5 mg/L and approximately 80% degradation at 15 mg/L.

Adsorption experiments conducted for 30 min showed limited Rhodamine B removal for both materials under the evaluated conditions. At a catalyst dosage of 0.4 g/L, RG-Sb_2_S_3_ achieved removals of 1.57 ± 0.47% and 3.61 ± 1.03% for initial Rhodamine B concentrations of 5 and 15 mg/L, respectively, while BPS reached 8.25 ± 0.14% and 7.55 ± 3.61%. Increasing the dosage to 0.8 g/L enhanced adsorption, with RG-Sb_2_S_3_ attaining removals of 6.19 ± 0.35% and 6.45 ± 3.56%, and BPS reaching 12.91 ± 3.21% and 5.37 ± 2.60% for 5 and 15 mg/L Rhodamine B, respectively. Overall, adsorption contributed less than 13% to Rhodamine B removal under the experimental conditions investigated, indicating that the observed dye removal was primarily associated with photocatalytic degradation. Furthermore, photolysis control experiments performed under identical irradiation conditions showed no measurable change in Rhodamine B concentration, confirming the negligible contribution of direct photolytic degradation. Nevertheless, further equilibrium adsorption studies are required for a comprehensive assessment of the adsorption behavior of the materials.

The effect of catalyst loading and initial dye concentration on photocatalytic performance can be interpreted considering both adsorption and photocatalytic processes. At 0.4 g/L, higher dye concentrations slightly enhanced removal, likely due to an increased mass-transfer driving force and greater interaction between dye molecules and available active sites [64,65]. In contrast, at 0.8 g/L, higher photocatalytic activity was observed at lower Rhodamine B concentrations, possibly due to improved active-site availability and reduced competition for adsorption. At higher dye concentrations, partial saturation of active sites, along with reduced light penetration and light attenuation by dye molecules, may limit photon absorption by the photocatalyst and decrease overall photocatalytic efficiency [64,65].

The degradation efficiency obtained at an initial Rhodamine B concentration of 5 mg/L (>98%) is within the range reported for advanced photocatalytic systems, including Bi_2_O_3_/Sb_2_S_3_ heterojunctions (98.2% at 0.3 g/L catalyst dosage) [20], g-C_3_N_4_ (98.7% at 1 g/L) [66], Ce-ZnO (97.72% at 1 g/L) [67], and MnO_2_/persulfate systems (97.41% at 0.8 g/L) [68]. Notably, BPS achieved comparable degradation efficiencies at a catalyst loading within the range commonly employed for these advanced systems. Direct comparisons with literature results should be interpreted with caution, as photocatalytic performance is strongly influenced by factors such as irradiation source and intensity, reactor configuration, reaction time, catalyst properties, catalyst dosage, and initial pollutant concentration.

**Figure 8:**
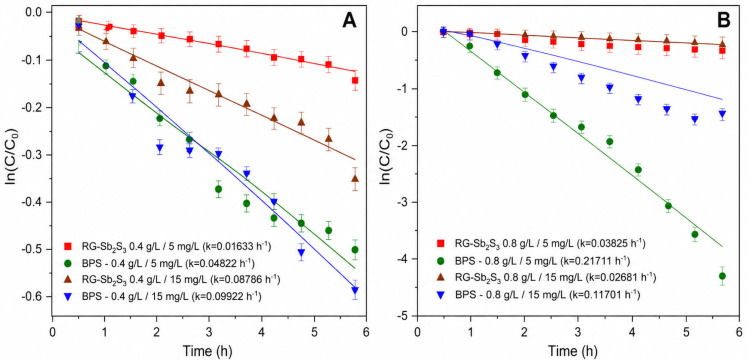
Pseudo-first-order kinetic modeling of Rhodamine B degradation under different catalyst loadings: (**A**) 0.4 g/L; (**B**) 0.8 g/L.

Rhodamine B degradation under solar irradiation followed a pseudo-first-order kinetic model based on Langmuir–Hinshelwood behavior. Linear fits of ln(C/C_0_) vs. time yielded apparent rate constants (*k*_app_) with R^2^ > 0.90, Table 2, indicating good agreement with the model. At a catalyst loading of 0.4 g/L, BPS exhibited higher *k*_app_ values than RG-Sb_2_S_3_ at all tested concentrations, with rate constants increasing with Rhodamine B concentrations from 5 to 15 mg/L, suggesting mass transfer limitations at low catalyst loading (Figure 8A). At 0.8 g/L and an initial Rhodamine B concentration of 5 mg/L, BPS exhibited the highest *k*_app_, more than five times higher than that of RG-Sb_2_S_3_. Increasing the dye concentration to 15 mg/L resulted in a reduction in *k*_app_ by approximately 50%, consistent with active site saturation typical of Langmuir–Hinshelwood kinetics (Figure 8B).

The influence of Rhodamine B concentration on the apparent reaction rate can be interpreted considering both kinetic and structural factors. At the lower catalyst loading (0.4 g/L), the increase in *k*_app_ with increasing dye concentration may indicate that the system was operating under mass-transfer constraints at the lowest pollutant concentration, where the probability of effective interactions between Rhodamine B molecules and photoactive sites was reduced. In contrast, at 0.8 g/L, the higher catalyst availability enhanced pollutant–catalyst interactions, and the decrease in *k*_app_ observed at 15 mg/L is consistent with Langmuir–Hinshelwood behavior, where the number of available active sites becomes insufficient relative to the pollutant load [69,70]. Additionally, higher dye concentrations can attenuate incident light through a Beer–Lambert effect, reducing photon penetration and limiting the activation of photocatalytic sites [71,72].

The structural characteristics of BPS may further contribute to its superior performance. Although the material exhibits a moderate specific surface area (2.99 m^2^ g^−1^), its combined micro- and mesoporous structure may promote Rhodamine B adsorption while facilitating mass transfer to photoactive sites, reducing diffusion limitations commonly associated with predominantly microporous materials [70,73]. Furthermore, the fibrous morphology observed by SEM provides accessible surface regions for pollutant interaction. The coexistence of multiple metal(loid) sulfide phases may also favor synergistic interfacial interactions that enhance photocatalytic activity, although dedicated charge-transfer studies would be required to directly verify this contribution [65,74].

A tentative photocatalytic degradation pathway can be proposed based on the characteristics of the recovered material and previous reports on Sb_2_S_3_/Bi_2_O_3_ photocatalytic systems. Under solar irradiation, the generation of photoinduced electron–hole pairs may promote the formation of reactive oxygen species, such as hydroxyl (•OH) and superoxide (•O_2_^−^) radicals, which have been widely reported as key oxidizing agents in dye degradation processes. These species can attack susceptible sites within the Rhodamine B molecule, particularly the N-ethyl substituents, leading to progressive de-ethylation and disruption of the conjugated chromophore responsible for its characteristic coloration. Further oxidation may subsequently affect the xanthene ring structure and promote cleavage of C–N bonds and aromatic moieties, resulting in the formation of smaller intermediate compounds and progressive mineralization. The proposed degradation behavior is consistent with pathways previously reported for Sb_2_S_3_/Bi_2_O_3_ photocatalysts [20]. Additionally, the slightly higher adsorption capacity observed for BPS may have increased the availability of Rhodamine B molecules at the catalyst surface, facilitating their subsequent photocatalytic transformation. Nevertheless, further studies involving radical scavenging experiments and complementary electrochemical analyses are required to experimentally validate the proposed mechanism.

The enhanced photocatalytic performance of BPS may be associated with its combined structural and optical properties. The hierarchical acicular–fibrillar morphology, together with its visible-light-responsive band gap, may facilitate improved light harvesting and contribute to the observed photocatalytic activity. However, given the complexity of the material and its origin from metallurgical effluents, the specific mechanisms governing charge separation and photocatalytic behavior require further investigation.

Nevertheless, the *k*_app_ values obtained for BPS (0.00195–0.0036 min^−1^) were lower than those reported for ZnO (0.0180–0.0343 min^−1^) [67,75], Ce-ZnO (0.0363 min^−1^) [67], g-C_3_N_4_ (0.0352 min^−1^) [66], Bi_2_O_3_/Sb_2_S_3_ heterojunctions (0.03149 min^−1^) [20], and Sb_2_S_3_/rGO composites (0.00839 min^−1^) [12]. Although these values are comparatively lower, it is important to consider that BPS was not synthesized through conventional controlled routes but was instead obtained through the recovery of metal(loid) sulfides from metallurgical effluents. Despite this, the material demonstrated measurable photocatalytic activity under solar irradiation, supporting its potential as a low-cost, waste-derived, and sustainable photocatalyst within a circular economy framework.

#### 2.2.2. Preliminary Reusability Assessment and Post-Reaction Characterization of BPS

A preliminary assessment of BPS reusability was conducted through three consecutive photocatalytic cycles. The objective was to examine potential morphological and compositional changes after reuse rather than to establish long-term catalyst stability.

**Figure 9:**
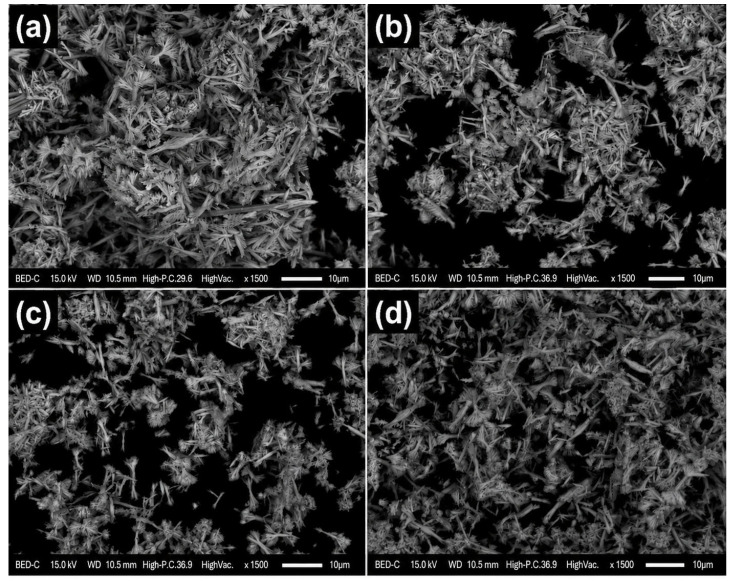
SEM micrographs in backscattered electron detector (BED) mode of polymetallic sulfide material (BPS): (**a**) before degradation cycles, (**b**) after the first degradation cycle, (**c**) after the second degradation cycle, and (**d**) after the third degradation cycle.

**Figure 10:**
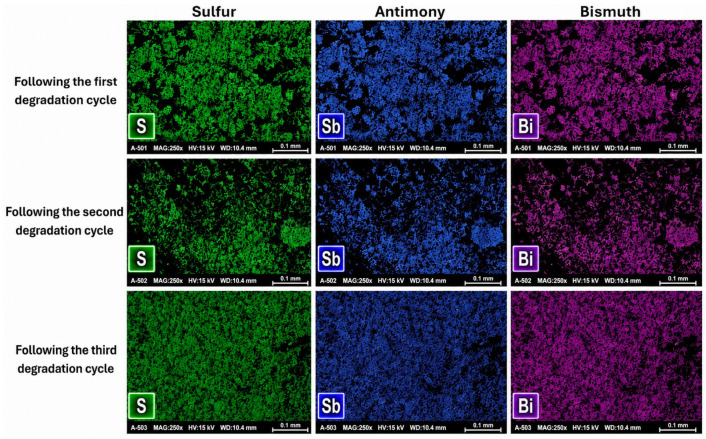
Post-degradation elemental mapping of biogenically recovered mixed metal(loid) sulfides (BPS) for antimony, sulfur, and bismuth.

The SEM analysis of BPS after three photocatalytic cycles is shown in Figure 9. Results indicate that the material retained its characteristic flower-like porous morphology, with no significant agglomeration or fracture observed after repeated use. SEM–EDS analyses additionally revealed a relatively uniform elemental distribution, suggesting limited phase segregation within the recovered material (Figure 10).

Particle size showed a slight decrease (~0.60 μm after three cycles), suggesting minor surface erosion during photocatalytic operation while preserving the overall structural integrity of the material. SEM–EDS analysis also revealed an increase in carbon content after the photocatalytic cycles (from 6.93% to 10.94%), likely associated with the adsorption of residual organic species or degradation intermediates onto the catalyst surface. Slight variations in chlorine content were similarly attributed to surface adsorption phenomena.

In contrast, sulfur and antimony contents remained relatively unchanged after repeated use, indicating good chemical stability of the sulfide-based material. Likewise, the stable bismuth content suggests preservation of the heterojunction-related structures that may contribute to the photocatalytic performance.

Furthermore, elemental mapping (Figure 10) confirmed a homogeneous distribution of Sb_2_S_3_-associated components, with no clear evidence of localized accumulation, phase segregation, or significant degradation after cycling.

Photocatalytic degradation of Rhodamine B was quantitatively determined during the first reuse cycle, reaching a removal efficiency of 64.26 ± 3.1%. The reusability assessment subsequently focused on evaluating the structural and compositional characteristics of the recovered photocatalyst through SEM–EDS analyses after successive cycles. In this regard, the material recovered from later cycles was primarily used for post-reaction morphological and elemental characterization to assess whether substantial changes occurred during repeated operation.

Overall, SEM–EDS and elemental mapping analyses demonstrated that BPS largely retained its morphological features and elemental distribution after three reuse cycles. The absence of major morphological alterations suggests that severe structural deterioration did not occur under the evaluated conditions. However, these observations should be regarded as a preliminary assessment of catalyst durability, and additional studies involving extended photocatalytic cycling and quantitative performance evaluation in each cycle are required to fully establish the long-term stability of the recovered material. Nevertheless, the results obtained here provide initial evidence that waste-derived polymetallic sulfides can remain active and structurally stable during repeated photocatalytic operation.

Further investigations should evaluate phase stability, surface chemical modifications, and potential metal leaching during extended operation through complementary characterization techniques. Additionally, the long-term reuse behavior of recovered polymetallic sulfides should be examined, including catalyst regeneration or reactivation strategies between photocatalytic cycles. Attention should be given to post-treatment conditioning procedures, as these may influence surface activity, sulfide phase stability, and overall photocatalytic performance upon successive reuse cycles.

#### 2.2.3. Implications and Future Perspectives for Biogenically Recovered Mixed Metal(Loid) Sulfides Photocatalysts

The results obtained in this study demonstrate the feasibility of recovering and valorizing mixed metal(loid) sulfides from metal-laden waste streams as photocatalytically active materials, highlighting the potential of integrating waste treatment and material generation within a circular and sustainable approach. Although the photocatalytic performance achieved under solar irradiation was promising, several challenges and opportunities for improvement remain.

Some limitations are intrinsically associated with metal sulfide photocatalysts, particularly their restricted ability to efficiently utilize the full solar spectrum, including limited absorption in the near-infrared (NIR) region [60]. In addition, the recovered materials obtained through biological precipitation processes may exhibit compositional heterogeneity and limited control over crystal growth, morphology, and particle size when compared with highly engineered synthetic photocatalysts.

Nevertheless, these biogenically recovered mixed metal(loid) sulfides represent an attractive platform for further optimization. Future studies could focus on controlling recovery and precipitation conditions to tailor morphology and composition, as well as exploring strategies such as selective doping, composite material formation, and heterostructure engineering to enhance charge separation and photocatalytic efficiency. The implementation of these strategies could further improve the photocatalytic behavior of recovered metal(loid) sulfides while retaining the sustainability benefits inherent to waste valorization processes.

An additional aspect that warrants further investigation is the environmental stability of the recovered material. Although elements associated with sulfur-containing phases are generally characterized by low solubility and have been widely investigated for reducing metal and metalloid mobility, the potential release of metal species during photocatalytic operation was not evaluated in the present study. Furthermore, polymetallic sulfides may undergo surface oxidation and transformation under irradiation and oxidative conditions, which could affect their environmental performance and durability during long-term operation. Therefore, future work should include leaching analyses and complementary stability assessments to assess metal release and establish the environmental safety of these materials during prolonged photocatalytic operation.

Overall, this work opens new perspectives for the development of functional photocatalytic materials derived from metallurgical residues through biologically assisted recovery routes, supporting the transition toward more circular strategies for waste management and environmental remediation.

## 3. Materials and Methods

### 3.1. Preparation of the Biogenically Recovered Mixed Metal(Loid) Sulfides (BPS)

The material used in this study was obtained by sulfide precipitation in a two-stage system coupled to the production of biogenic sulfide via sulfate reduction in a UASB reactor as described preciously in [26]. The metallurgical effluent used originated from an industrial metallurgical refining process associated with the extraction of base and precious metals with the composition described in [26].

Daily batch experiments were conducted to accumulate sufficient material for physicochemical characterization and photocatalytic evaluation.

Following precipitation, the wet material was separated from the liquid phase by natural sedimentation and subsequently dried for 24 h at room temperature under anaerobic conditions without external heating. This post-treatment procedure was selected to minimize oxidation of the sulfide phases, preserve the crystalline structure, and avoid phase transformations or the formation of amorphous byproducts that could affect the optical and photocatalytic properties of the recovered material [26]. After drying, the solid was stored in airtight containers protected from atmospheric oxygen and moisture until further characterization and photocatalytic testing.

### 3.2. Characterization Methods

The crystal structure was analyzed by X-ray diffraction (XRD, Bruker AXS D8 Advance, Bruker Corporation, Karlsruhe, Germany, Cu Kα, λ = 1.5406 Å, 40 mA, 45 kV) in the range of 2θ = 10–80°. Surface morphology and elemental composition were evaluated by scanning electron microscopy coupled to X-ray energy dispersive spectroscopy (SEM–EDS, Hitachi TM3030plus Hitachi High-Technologies Corporation, Tokyo, Japan, with Bruker detector), following the EPA/600/R-02/070 standard [76]. Prior to analysis, the biogenically precipitated sulfides (BPS) were dried under anaerobic conditions as described previously. The dried material was gently homogenized to ensure representative sampling while preserving its original morphology and then mounted directly onto carbon adhesive tape attached to aluminum sample holders. No washing, chemical treatment, or aggressive grinding was performed before analysis to avoid alterations to the surface characteristics of the recovered sulfides. Samples were handled with minimal exposure to ambient air during mounting and immediately transferred to the microscope chamber for characterization.

The specific surface area was determined by N_2_ adsorption at 77 K (BET, Micromeritics Gemini VII 2390t, Micromeritics Instrument Corporation, Norcross, GA, USA). The samples were degassed under vacuum at 150 °C for 12 h prior to analysis.

The band gap energy (*E*_g_) was estimated by diffuse reflectance UV–Vis spectroscopy (DRS) using a Perkin Elmer Lambda 20 spectrophotometer equipped with a solid-sample accessory (PerkinElmer, Waltham, MA, USA). The spectra were recorded in the range of 200–800 nm, and the reflectance data were transformed using the Kubelka–Munk function [47,48,77]:

(1) $$F\left(R_{\infty }\right)=\frac{(1-R{)}^{2}}{2R}$$

where *R* is the relative diffuse reflectance. Subsequently, the band gap energy was estimated using the Tauc method according to the following relationship:

$${\left(F\left(R\right)h\nu \right)}^{n}=A\left(h\nu -E_{g}\right)$$

where *hν* represents the photon energy and *n* depends on the type of electronic transition considered. Due to the heterogeneous nature of the BPS material, both allowed direct transitions (*n* = 1/2) and allowed indirect transitions (*n* = 2) were evaluated since for Sb_2_S_3_-based materials, the literature reports both transition behaviors depending on structural factors such as crystallinity, defects, composition, and synthesis method [48].

The *E*_g_ values were estimated by linear extrapolation of the most well-defined region near the optical absorption edge in the Tauc plots [49,50].

Infrared spectra were obtained by FTIR spectroscopy (Thermo Scientific Nicolet iS50, Thermo Fisher Scientific, Waltham, MA, USA) in transmission mode, using KBr pellets over the range of 4000–400 cm^−1^ with a resolution of 4 cm^−1^.

The surface chemical composition and oxidation states of the elements present in the material were analyzed by X-ray photoelectron spectroscopy (XPS). XPS analysis was performed using a Perkin-Elmer PHI 5100 spectrometer (PerkinElmer, Waltham, MA, USA) with Mg Kα radiation. Measurements were conducted under high vacuum (~2 × 10^−8^ Torr). Binding energies were calibrated using the C 1*s* peak at 284.8 eV. Data processing included FFT smoothing and Tougaard background subtraction, and spectra were deconvoluted using Gaussian functions based on NIST reference data [17].

### 3.3. Photocatalytic Degradation Experiments

Rhodamine B (reagent grade, Sigma-Aldrich, St. Louis, MO, USA) was used as a model contaminant. A stock solution with a concentration of 100 mg/L was prepared in deionized water, from which working solutions were obtained. Photocatalytic activity was evaluated at initial concentrations of 5 and 15 mg/L, using catalyst loadings of 0.4 and 0.8 g/L. Two materials were tested: reagent-grade Sb_2_S_3_ (RG-Sb_2_S_3_) (Sigma-Aldrich, St. Louis, MO, USA) and the biogenically recovered mixed metal(loid) sulfides (BPS).

Prior to irradiation, the reaction suspensions were subjected to a 30 min dark adsorption period to assess the contribution of dye adsorption in the absence of light. Subsequently, photocatalytic experiments were carried out under natural solar irradiation, always within the same daily time interval to minimize variations in solar intensity. Based on irradiance measurements recorded during the experimental period, the average solar irradiance was 782 ± 64 W m^−2^ (Figure S5).

All experiments were conducted under continuous stirring (300 rpm) for 300 min, and aliquots were collected every 30 min to monitor Rhodamine B degradation. Photolysis controls were carried out under the same irradiation conditions, reaction times, and Rhodamine B concentrations, but in the absence of catalyst. All experiments were performed in triplicate.

Rhodamine B concentration was determined by UV–Vis spectrophotometry (HACH DR 5000, Hach Company, Loveland, CO, USA) at λ_max_ = 555 nm using a calibration curve. Degradation efficiency was calculated as the relative decrease in concentration over time [12].

(2) $$\%DE=\frac{A_{0}-A_{t}}{A_{0}}\times 100\;$$

The kinetics of photocatalytic degradation were evaluated using the Langmuir–Hinshelwood model, assuming pseudo-first-order behavior, expressed in its linear form as:

(3) $$\ln\left(\frac{C}{C_{0}}\right)=-kt\;$$

where *k* is the apparent reaction rate constant (*k*_app_), *C*_0_ is the initial concentration, and *C* is the concentration at time *t*.

Catalyst reusability was evaluated by recovering the solid, drying, and reusing it in subsequent cycles with 5 mg/L Rhodamine B.

## 4. Conclusions

The biogenically recovered mixed metal(loid) sulfides (BPS), characterized by a predominantly crystalline structure, fibrous morphology, and a narrow direct band gap (1.306 eV), exhibited effective visible-light absorption, supporting their applicability as photocatalysts for solar-driven water treatment processes.

Under solar irradiation, BPS demonstrated higher photocatalytic activity than reagent-grade Sb_2_S_3_ (RG-Sb_2_S_3_) during Rhodamine B degradation experiments. Under the optimal conditions evaluated (0.8 g L^−1^ catalyst and 5 mg L^−1^ Rhodamine B), degradation efficiencies above 98% were achieved, while the apparent reaction rate constant was more than five times higher than that of RG-Sb_2_S_3_. These results highlight the feasibility of obtaining effective photocatalytic materials from metallurgical effluents through recovery-based approaches.

The degradation process followed a pseudo-first-order kinetic model consistent with Langmuir–Hinshelwood behavior (R^2^ > 0.90). Variations in photocatalytic performance were influenced by catalyst loading and pollutant concentration, with lower apparent rate constants at higher dye concentrations being associated with active-site saturation and light attenuation effects.

A preliminary reusability assessment and post-reaction characterization indicated that BPS retained its morphological and compositional integrity after three photocatalytic cycles, with no significant evidence of structural deterioration. Although additional quantitative reuse studies are required to establish long-term photocatalytic stability, the results demonstrate the robustness of the recovered material under the evaluated conditions.

Overall, this study demonstrates that biogenically recovered mixed metal(loid) sulfides can serve as effective waste-derived photocatalysts for solar-driven pollutant degradation. These findings support the integration of metallurgical waste valorization, resource recovery, and advanced oxidation processes within more sustainable and circular approaches to wastewater treatment.

## Figures and Tables

**Table 1:** Textural properties of RG-Sb_2_S_3_ and BPS determined by N_2_ physisorption (BET).

Material	Surface Area (m^2^/g)	Total Pore Volume (cm^3^/g)	Average Pore Diameter (nm)
Reagent-grade Sb_2_S_3_ (RG-Sb_2_S_3_)	0.338 ± 0.02	0.0002 ± 0.002	2.137 ± 0.47
BPS	2.992 ± 0.13	0.0001 ± 0.010	2.118 ± 0.28

**Table 2:** Pseudo-first-order kinetic parameters obtained for the different experimental conditions.

Photocatalyst	Photocatalyst Concentration (g/L)	Contaminant Concentration (mg/L)	Apparent Rate Constant (*k*_app_), h^−1^	Coefficient of Determination (R^2^)
RG-Sb_2_S_3_	0.4	5	0.01633	0.9329
		15	0.08786	0.9182
BPS		5	0.04822	0.9071
		15	0.09922	0.9450
RG-Sb_2_S_3_	0.8	5	0.03825	0.9333
		15	0.02681	0.9713
BPS		5	0.21711	0.9914
		15	0.11701	0.9537

## Data Availability

Data Availability upon request.

## Supplementary Materials

The following supporting information can be downloaded at: https://www.mdpi.com/article/10.3390/ijms27135689/s1.
