# Supplementary material for: Solar-Driven Rhodamine B Degradation Using Biogenically Recovered Mixed Metal(Loid) Sulfides Derived from Metallurgical Waste
Source: Int J Mol Sci. 2026 Jun 24;27(13):5689. doi: 10.3390/ijms27135689 (PMC13361936; doi:10.3390/ijms27135689)
Supplement: Supplementary file 1 [file ijms-27-05689-s001.zip › ijms-4373243-supplementary.pdf]

Supporting information

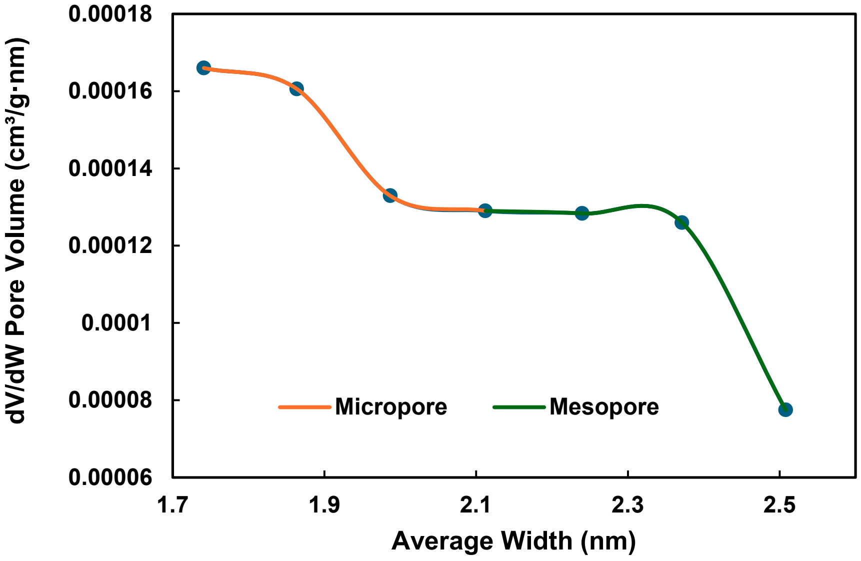

Figure S1. Pore size distribution curves of the BPS material.

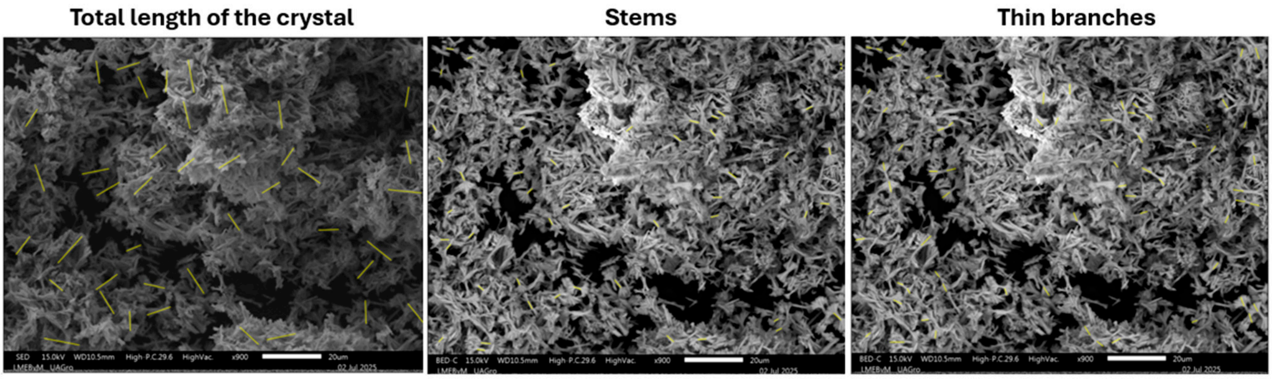

Figure S2. Morphometric analysis corresponding to biogenically recovered mixed meal(loid) sulfides (BPS).

Table S1. Morphometric analysis corresponding to biogenically recovered mixed meal(loid) sulfides (BPS).

Structures	Measurements	Average Measurements (um)	Standard Deviation
Total length of the crystal	146	7.397	0.965
Stems or columns	146	2.379	0.484
Thin branches	146	2.583	0.638

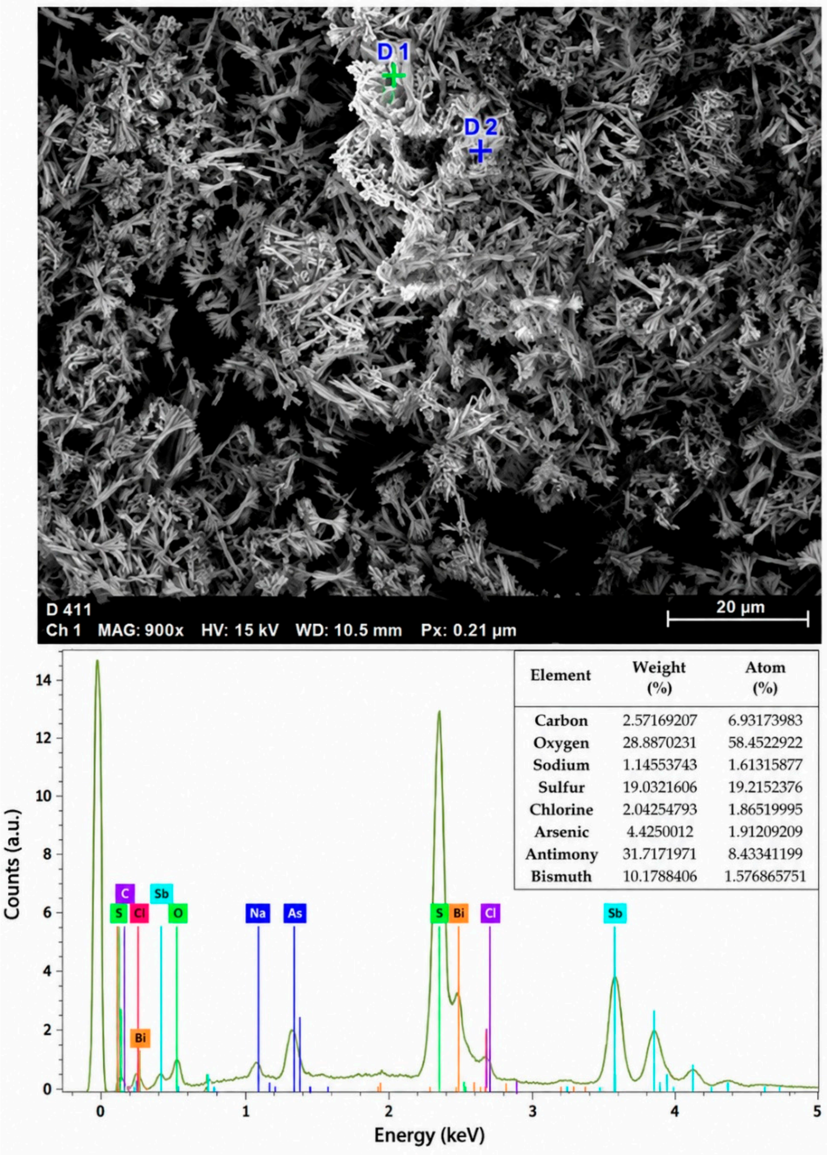

Element	Weight (%)	Atom (%)
Carbon	2.57169207	6.93173983
Oxygen	28.8870231	58.4522922
Sodium	1.14553743	1.61315877
Sulfur	19.0321606	19.2152376
Chlorine	2.04254793	1.86519995
Arsenic	4.4250012	1.91209209
Antimony	31.7171971	8.43341199
Bismuth	10.1788406	1.576865751

Figure S3. Image of the EDS analysis corresponding to biogenically recovered mixed meal(loid) sulfides (BPS).

Table S2. Parameters of the linear regression used for direct and indirect band gap estimation of BPS.

Equation	y = a + b*x	y = a + b*x	y = a + b*x	y = a + b*x
**Plot**	Tangent, BPS n=2	Linear Fit, BPS n=2	Tangent, BPS n=1/2	Linear Fit, BPS n=1/2
**Intercept**	-27.20743 ± 0.14988	-27.20743 ± 1.40376E-14	-5.43631 ± 0.02649	-5.43631 ± 6.79255E-15
**Slope**	18.40966 ± 0.08858	18.40966 ± 8.2274E-15	4.17858 ± 0.01837	4.17858 ± 4.69207E-15
**R-Square (COD)**	0.99265	1	0.99521	1
**Adj. R-Square**	0.99262	1	0.99519	1

Table S3. Parameters of the linear regression used for direct and indirect band gap estimation of RG-Sb_2S_3.

Equation	y = a + b*x	y = a + b*x	y = a + b*x	y = a + b*x
**Plot**	Tangent, RG-Sb2S3 n=2	Linear Fit, RG-Sb2S3 n=2	Tangent, RG-Sb2S3 n=1/2	Linear Fit, RG-Sb2S3 n=1/2
**Intercept**	-333.77116 ± 1.80757	-333.77116 ± 6.05753E-13	-12.84863 ± 0.10627	-12.84863 ± 2.02899E-14
**Slope**	201.22957 ± 1.03311	201.22957 ± 3.45696E-13	8.53087 ± 0.06352	8.53087 ± 1.21068E-14
**R-Square (COD)**	0.99656	1	0.99214	1
**Adj. R-Square**	0.99653	1	0.99208	1

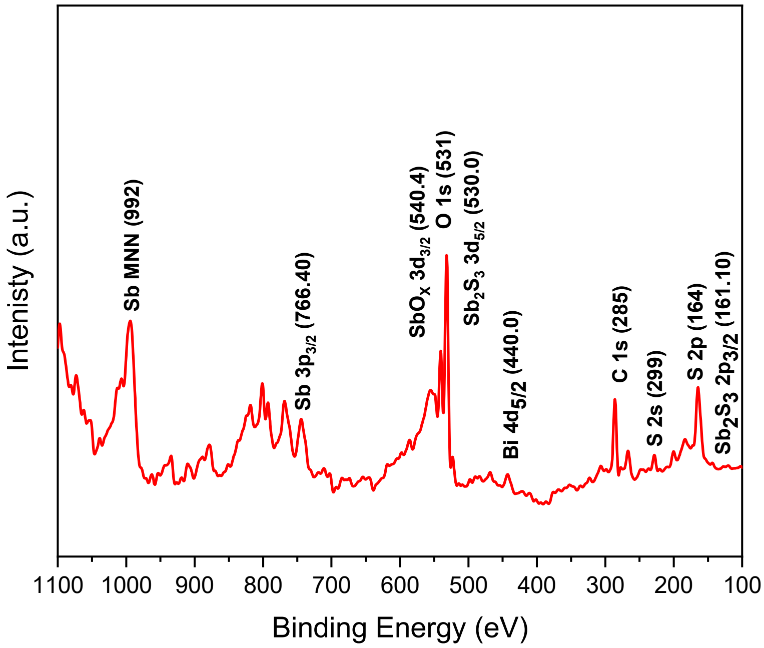

Figure S4. Survey XPS spectrum of biogenically recovered mixed meal(loid) sulfides (BPS).

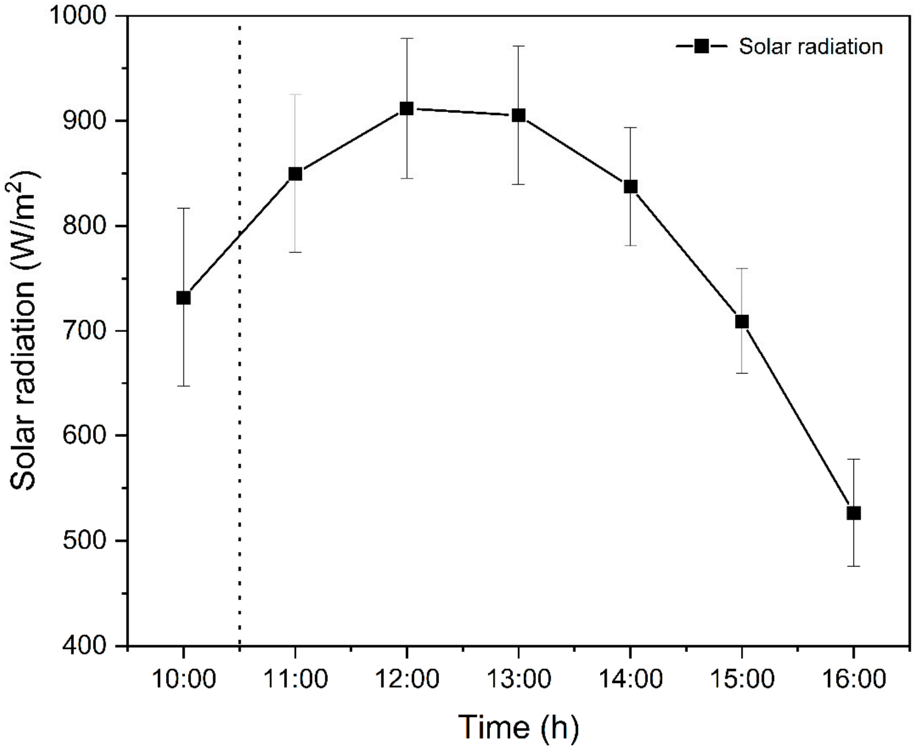

Figure S5. Solar irradiance recorded during the experimental period used for the photocatalytic experiments under natural solar irradiation (10:00–16:00 h). The average irradiance was 782 ± 64 W m^{-2}.
